# Diagnostic Uncertainty in Rapidly Progressive Brainstem Lesions: A Radiological Diagnosis of Probable CNS Relapse of Diffuse Large B-cell Lymphoma

**DOI:** 10.7759/cureus.115416

**Published:** 2026-08-29

**Authors:** Yik Shun Eason Chan, Pranav Santhosh Kumar, Tom Bull, Brett Ibbeson

**Affiliations:** 1 Radiology, West Suffolk Hospital, Bury St. Edmunds, GBR; 2 Haematology, West Suffolk Hospital, Bury St. Edmunds, GBR

**Keywords:** chorea-ballismus, cns lymphoma, cns lymphoma radiological appearance, diffuse large b cell lymphoma (dlbcl), diffusion restriction (dwi), mri, non hodgkin's lymphoma, relapse lymphoma

## Abstract

We present the case of a 78-year-old woman with a history of diffuse large B-cell lymphoma (DLBCL), previously treated as per the REMoDL-B (Randomised Evaluation of Molecular-Guided Therapy for Diffuse Large B-cell Lymphoma With Bortezomib) protocol with rituximab, bortezomib, cyclophosphamide, doxorubicin, vincristine, and prednisolone, alongside intrathecal methotrexate for CNS prophylaxis, who presented 10 years after achieving systemic remission with a 15-day history of hyperactivity, choreiform movements, pressured speech, and incontinence. Initial differentials included mania, hyperactive delirium, and infective or autoimmune encephalitis. An extensive autoimmune, infective, and paraneoplastic screen, together with cerebrospinal fluid (CSF) cytology and flow cytometry, was unrevealing.

Initial magnetic resonance (MR) imaging showed bilateral midbrain and pons hyperintensity on T2-weighted fluid-attenuated inversion recovery (T2-FLAIR), with no evidence of restricted diffusion, a pattern that led the clinical team away from the diagnosis of central nervous system (CNS) lymphoma relapse and towards an inflammatory or infective process. Repeat MR imaging of the head was performed after the patient deteriorated clinically despite empirical treatment for infective encephalitis with intravenous ceftriaxone and aciclovir. This demonstrated marked radiological progression, with subsequent contrast-enhanced MRI revealing homogeneous enhancement extending to the bilateral thalami and basal ganglia, with newly developed diffusion restriction, a pattern substantially more typical of CNS lymphoma. Positron emission tomography-computed tomography (PET-CT) confirmed metabolically active disease at corresponding sites but identified no extracranial target for biopsy; the deep bilateral location of the lesion was deemed too high risk to allow stereotactic sampling.

In the absence of histological confirmation, the case was discussed at the regional neuro-oncology multidisciplinary team (MDT) meeting, where isolated CNS relapse of DLBCL was adopted as the most probable diagnosis on radiological and clinical grounds alone. Given an Eastern Cooperative Oncology Group (ECOG) performance status of 4, the patient was not a candidate for treatment and was discharged on a palliative pathway.

This case highlights three key learning points: CNS relapse of DLBCL should remain an active diagnostic consideration regardless of the duration of prior remission; the initial lack of diffusion restriction on early MR imaging is not sufficient to exclude CNS lymphoma; and, when tissue diagnosis is unattainable, early neuro-oncology MDT involvement is essential to reach a working diagnosis on radiological and clinical grounds before the window for potential treatment closes.

## Introduction

Diffuse large B-cell lymphoma (DLBCL) is an aggressive but often curable B-cell malignancy and the most common subtype of non-Hodgkin lymphoma (NHL) [1]. Despite advances in chemoimmunotherapy, relapse in the central nervous system (CNS) remains one of the most feared and therapeutically challenging complications of DLBCL, occurring in approximately 0.6-10% of patients within two years, depending on baseline risk stratification [2].

When CNS relapse occurs, the prognosis is poor, with median overall survival typically measured in months [3]. Treatment options are substantially constrained by patient age, performance status, and the neurotoxicity of CNS-penetrant agents. Late CNS relapse of NHL, occurring years after initial systemic remission, is less common than early relapse but well documented [4] and may be particularly challenging to diagnose because the clinical context of active systemic lymphoma is absent, reducing the index of suspicion.

The clinical presentation of CNS relapse of DLBCL is highly variable, reflecting the wide range of intracranial sites that may be involved. Focal neurological deficit is the most common presenting feature, but behavioural changes, neuropsychiatric disturbances, and movement disorders, including choreiform movements and mania-like features, have documented associations with CNS lymphoma and may lead clinicians towards a primary psychiatric or neurodegenerative differential before malignancy is considered [5,6]. This diagnostic challenge is compounded by the radiological overlap between CNS lymphoma and its inflammatory and infective mimics, including autoimmune encephalitis and infective rhombencephalitis, which can produce broadly similar patterns of thalamic and brainstem signal abnormality on non-contrast magnetic resonance imaging (MRI). Restricted diffusion on MRI, which reflects impaired movement of water molecules within densely packed tissue and appears as high signal on diffusion-weighted imaging with corresponding low signal on the apparent diffusion coefficient map, is considered characteristic of CNS lymphoma owing to its high tumour cellularity. However, it is not invariably present; reported rates of restricted diffusion in biopsy-proven CNS lymphoma range from approximately 72% to 90% across small imaging series [7,8]. Its absence therefore does not exclude the diagnosis and should not be used in isolation to do so.

To our knowledge, the specific combination of choreiform movements, mania-like neuropsychiatric features, and bilateral brainstem lesions as a presenting constellation of CNS relapse of DLBCL appears to be rarely reported, making this case a valuable contribution to the literature both clinically and radiologically. We present it to highlight three key learning points. First, an atypical constellation of choreiform movements and neuropsychiatric symptoms in a patient with prior DLBCL should prompt a high index of suspicion for CNS relapse, regardless of the absence of more classical clinical or radiological features or the duration of remission. Second, the lack of diffusion restriction on the initial MRI is not sufficient on its own to exclude CNS lymphoma on a non-contrast scan; short-interval repeat contrast-enhanced studies should be considered when suspicion for CNS lymphoma is high. Third, when lesion location or patient frailty precludes tissue diagnosis, early neuro-oncology MDT involvement is essential to allow a working diagnosis to be reached on radiological and clinical grounds alone, ensuring that the window for potential treatment is not missed.

## Case presentation

A 78-year-old woman presented with a history of DLBCL diagnosed in 2014, at which time the disease was confined to the left breast and left axillary lymph nodes (Ann Arbor stage IIE). She was treated as per the REMoDL-B (Randomised Evaluation of Molecular-Guided Therapy for Diffuse Large B-cell Lymphoma With Bortezomib) protocol [9] with rituximab, bortezomib, cyclophosphamide, doxorubicin, vincristine, and prednisolone, alongside intrathecal methotrexate for CNS prophylaxis. Her retrospectively calculated CNS-IPI (Central Nervous System International Prognostic Index) score was 2, placing her in the intermediate-risk group. Two variables were present at diagnosis: age over 60 and elevated lactate dehydrogenase. Treatment was completed in February 2015, with complete metabolic remission confirmed on interim assessment in April 2015. She remained in continuous remission thereafter. The interval from documented complete metabolic remission to the present presentation was 10 years. Other diagnoses included depression managed with sertraline and a right frontal infarct identified in 2023.

On presentation, the chief complaints included hyperactivity, choreiform involuntary movements, high-pitched pressured speech, poor coordination, and two episodes of both faecal and urinary incontinence over the preceding two weeks. There was no reported confusion or fever. Collateral history indicated a longer background of weight loss over the preceding months.

Neurological examination revealed bilateral large-amplitude involuntary movements that were ballismus-like in quality. No deficits in power, reflexes, or sensation were found in any of the four limbs, and no facial droop, gaze paresis, or pupillary abnormalities were detected. The patient was able to stand and walk with a steady gait.

On admission, systolic hypotension and bradycardia were observed, and blood tests showed mild anaemia, as demonstrated in Tables 1, 2. LDH was not measured on presentation.

**Table 1:** Initial blood tests Blood tests were performed in the emergency department at presentation. Results were within normal limits except for mild anaemia. No abnormality was identified to account for the presenting neurological and neuropsychiatric symptoms. Reference ranges are those of the reporting laboratory.

Investigation	Result	Reference Range
Haemoglobin (Hb)	113 g/L	115-165 g/L
White blood cell count	6.1 × 10⁹/L	4 × 10⁹/L to 11 × 10⁹/L
C-reactive protein (CRP)	<1.0 mg/L	<5mg/L
Sodium	136 mmol/L	133-146 mmol/L
Potassium	4.5 mmol/L	3.5-5.3 mmol/L
Free thyroxine (Free T4)	12.8 pmol/L	10.5-22 pmol/L
Thyroid stimulating hormone (TSH)	1.27 mIU/L	0.27-4.2 mIU/L
Adjusted calcium	2.24 mmol/L	2.2-2.6 mmol/L
Inorganic phosphate	0.96 mmol/L	0.8-1.5 mmol/L
Total serum bilirubin	8 µmol/L	<20 µmol/L
Serum alanine aminotransferase (ALT)	12 U/L	<33 U/L
Serum alkaline phosphatase (ALP)	71 U/L	30-130 U/L
Serum albumin level	44 g/L	35-50 g/L
Serum globulin level	20 g/L	18-31 g/L

**Table 2:** Initial vital signs Observations recorded at initial assessment. The patient was afebrile, with systolic hypotension and bradycardia. There was no other haemodynamic feature to suggest sepsis or an acute infective process at presentation.

Vital Sign	Result	Reference Range
Body temperature	36.5°C	36.1-37.5°C
Systolic blood pressure	97 mmHg	111-140 mmHg
Diastolic blood pressure	60 mmHg	60-90 mmHg
Heart rate	53 beats/minute	60-110 beats/minute
Oxygen saturation	95%	>94%
Respiratory rate	18 breaths/minute	14-20 breaths/minute

Initial investigations

Neurology and Psychiatry were consulted at this point, with a plan to start intravenous antibiotics and aciclovir, discontinue sertraline in case of mania, and initiate a comprehensive workup, as described in Table 3, to identify an organic cause for the presentation. Cerebrospinal fluid analysis demonstrated mildly elevated protein at 0.54 g/L (reference range: 0.15-0.40 g/L), with normal glucose and insufficient cellularity for flow cytometric analysis. This pattern was non-specific and did not discriminate between infective, inflammatory, and neoplastic aetiologies. No investigation in this panel identified a unifying cause for the clinical findings. 

**Table 3:** Panel of initial investigations Summary of serological, cerebrospinal fluid and radiological investigations undertaken to identify an infective, inflammatory or neoplastic cause. Ab: antibody; ANA: antinuclear antibody; CASPR2: contactin-associated protein-like 2; CSF: cerebrospinal fluid; CT-NCAP: computed tomography of the neck, chest, abdomen, and pelvis; CV2: collapsin response mediator protein 5 antibody; HIV: human immunodeficiency virus; HU: anti-Hu antibody (anti-neuronal nuclear antibody type 1); IgA: immunoglobulin A; JC virus: John Cunningham virus; L: litre; LGI1: leucine-rich glioma-inactivated 1; MA2: anti-Ma2 antibody; NMDA-R: N-methyl-D-aspartate receptor; p-ANCA: perinuclear anti-neutrophil cytoplasmic antibody; PCR: polymerase chain reaction; RI: anti-Ri antibody (anti-neuronal nuclear antibody type 2); TRAb: thyroid-stimulating hormone receptor antibody; tTG-IgA: tissue transglutaminase-immunoglobulin A.

Investigation	Result
Autoimmune encephalitis panel	Negative for NMDA-R, LGI1, CASPR2
Vasculitis screen	Negative for ANA, proteinase 3 Ab, myeloperoxidase Ab, weakly positive for p-ANCA
Paraneoplastic and thyroid antibodies	Negative for CV2, MA2, HU, RI, TRAb, thyroid peroxidase Ab
Lumbar puncture	Elevated protein 0.54 g/L (reference range 0.15 g/L-0.4 g/L). Normal glucose level. Negative for CSF viral PCR, microscopy/culture/sensitivity and cytology. White cell count 2 x10⁶/L. Red cell count 0 x 10⁶/L
CSF Flow cytometry	Non-diagnostic as sample sent had no white cells to be analysed
Infective screen	Negative for JC virus, syphilis, HIV, hepatitis B and C
Metabolic screen	Negative for copper and caeruloplasmin
CT-NCAP	No evidence of lymphadenopathy or extranodal disease
Coeliac serology	Negative for tTG-IgA, normal total IgA level

Taken together, this panel of investigations was unrevealing. It did not support an infective, autoimmune, or paraneoplastic process, nor did CT of the neck, chest, abdomen, and pelvis identify evidence of systemic relapse of lymphoma. With no positive supporting laboratory or cerebrospinal fluid findings, serial imaging became the principal means by which the differential diagnosis would ultimately be resolved.

Over the course of subsequent weeks, despite empirical intravenous (IV) ceftriaxone and aciclovir, the patient's clinical status deteriorated significantly, with increasing limb weakness and progression to bed-bound status and an ECOG performance status of 4.

Initial imaging

Computed tomography (CT) imaging of the head performed on admission showed no acute intracranial abnormality, with an area of encephalomalacia consistent with the previously known right anterior superior frontal gyrus infarct. Non-contrast MRI of the brain was performed shortly afterwards; gadolinium administration was not possible owing to the patient's involuntary movements. The scan demonstrated extensive T2-weighted fluid-attenuated inversion recovery (T2-FLAIR) hyperintensity affecting much of the midbrain and pons and extending to the cerebral peduncles. Diffusion-weighted imaging (DWI) on this initial scan showed no clear restricted diffusion, as shown in Figure 1, a feature interpreted as atypical for CNS lymphoma; the working differential at this point therefore favoured rhombencephalitis over CNS lymphoma.

**Figure 1:**
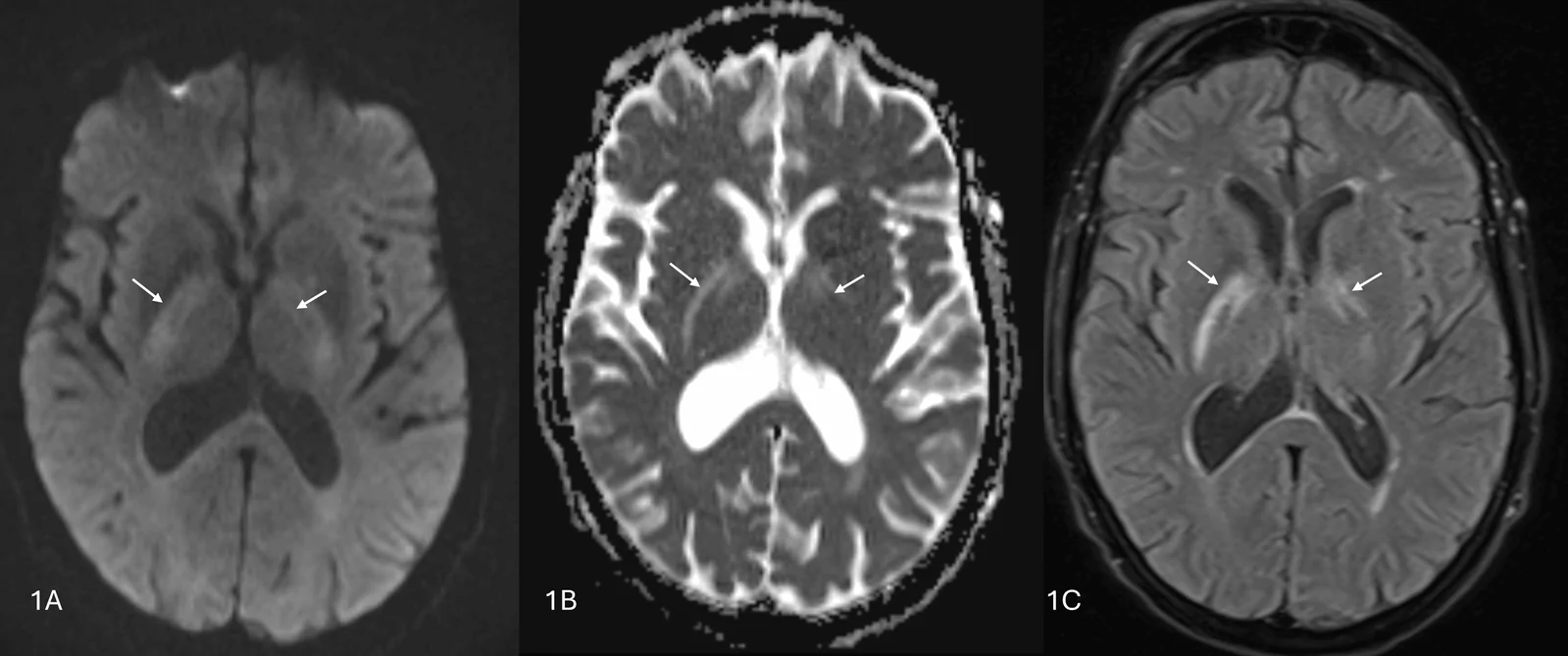
Initial non-contrast MRI of the head A-C: Initial non-contrast MRI showing T2-fluid-attenuated inversion recovery (T2-FLAIR) hyperintensity affecting bilateral thalamocapsular regions (arrows), but no clear restricted diffusion as commonly observed in lymphoma. A: Diffusion-weighted imaging (DWI). B: Corresponding apparent diffusion coefficient (ADC) map. C: T2-FLAIR sequence.

Serial MRI showing emergent diffusion restriction and contrast-enhanced MRI guiding the diagnosis of isolated CNS relapse of DLBCL

A repeat non-contrast MRI of the brain performed two and a half weeks after admission demonstrated marked radiological progression despite empirical treatment for infective encephalitis. Extensive FLAIR signal abnormality now extended to involve the cerebral peduncles, pons, right middle cerebellar peduncle, and bilateral basal ganglia, with inferior extension into the dorsal pontine tegmentum and right deep cerebellar white matter and anterior involvement of the optic tracts. Patchy diffusion restriction, predominantly within the deep thalamic regions, had not been seen on the initial study but was clearly more evident on this repeat scan, as shown in Figure 2. CNS lymphoma had initially been considered less likely based on the lack of diffusion restriction and only became the presumed diagnosis once this finding became more pronounced on MRI. 

**Figure 2:**
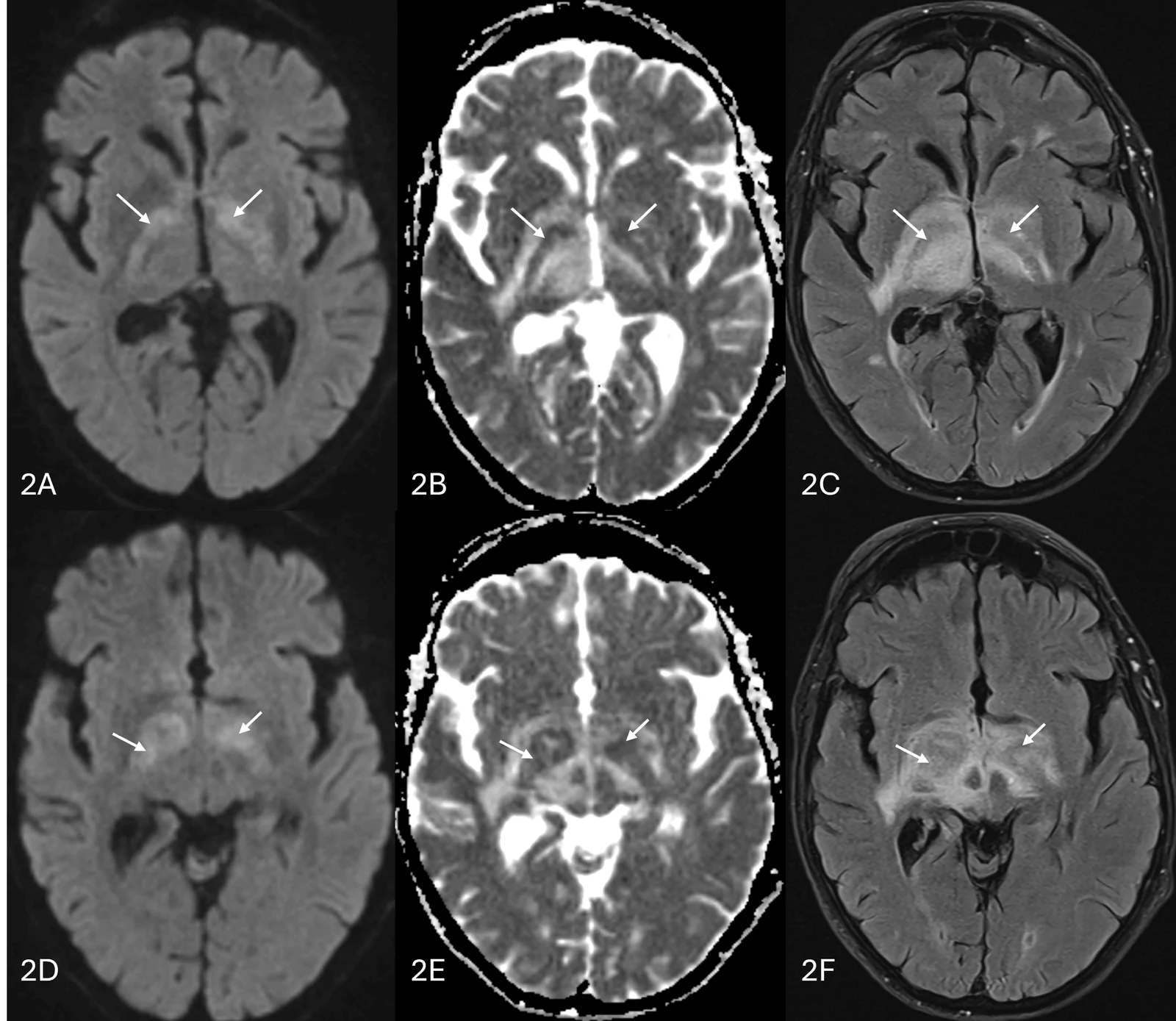
Repeat non-contrast MRI 2.5 weeks after initial imaging A-C: Progression of oedema as evidenced by more extensive FLAIR hyperintensity and new, patchy restricted diffusion suggestive of CNS lymphoma, demonstrated on the same plane as Figure 1. D-F: Slightly caudal plane, demonstrating more obvious restricted diffusion as evidenced by DWI high signal and corresponding ADC low signal. A and D: Diffusion weighted imaging (DWI). B and E: Corresponding apparent diffusion coefficient (ADC) map. C and F: T2 fluid-attenuated inversion recovery (FLAIR) sequence. Arrows pointing to patchy areas of new restricted diffusion not previously seen, potentially reflecting increased cellularity.

A contrast-enhanced MRI performed three days later (Figure 3) demonstrated extensive T2-FLAIR hyperintensity involving both basal ganglia, the thalami, midbrain, and pons. The distribution of this widespread change appeared similar to the previous scan. In addition, two discrete foci of uniform, avid post-contrast enhancement were identified: one in the anterior midbrain, corresponding to central areas of relatively low FLAIR signal with a T2-FLAIR hyperintense rim, and a second within the left hemipons. Restricted diffusion was reported at the right anterior midbrain, which appeared swollen relative to the left. ADC values were not quantified, and susceptibility-weighted sequences were not acquired. This combination of uniform, avid enhancement within an area of extensive FLAIR change, with emergent diffusion restriction, was considered more typical of CNS lymphoma than the earlier appearances and represented the pivotal imaging findings in establishing the working diagnosis.

**Figure 3:**
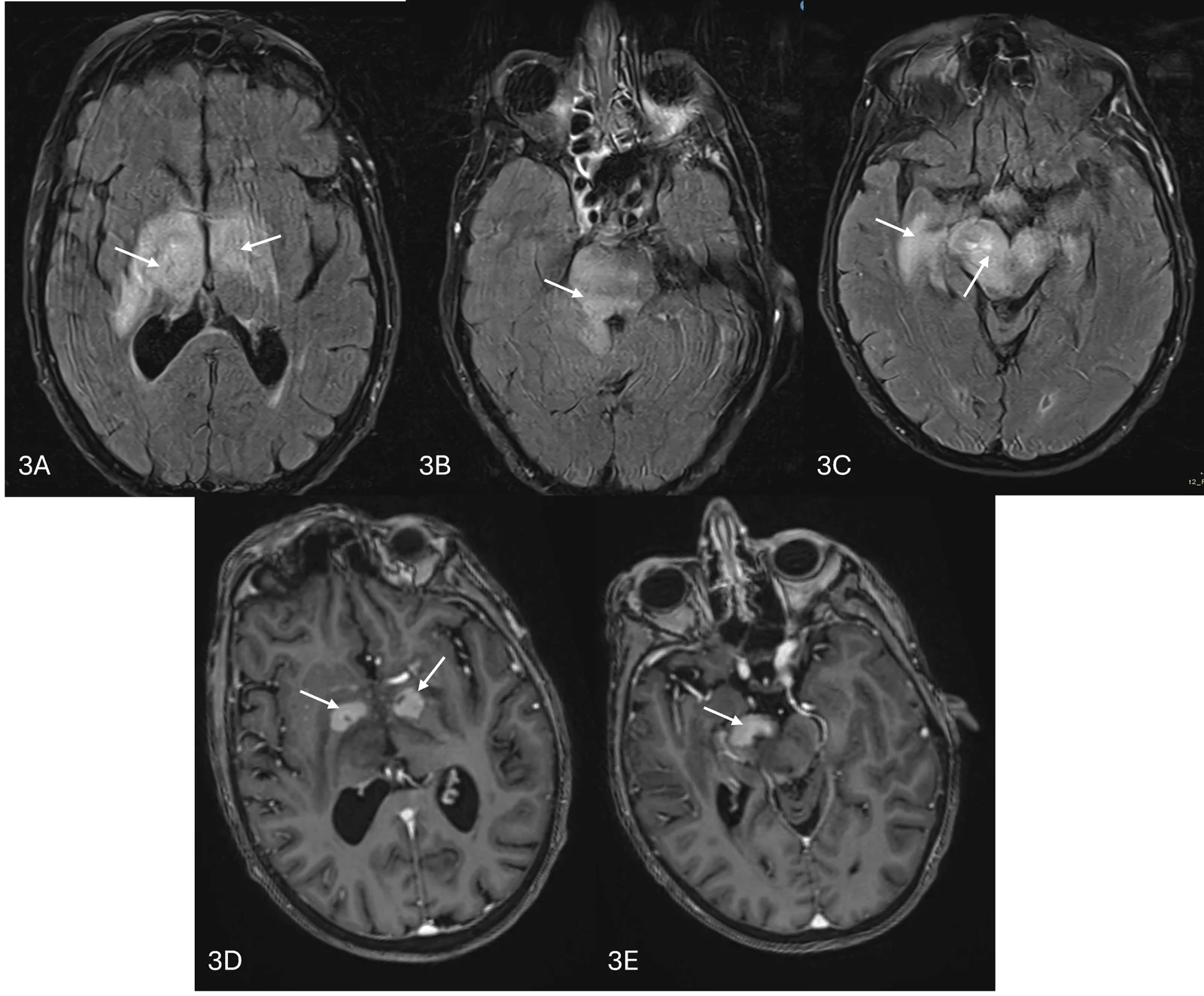
Repeat contrast-enhanced MRI of the head taken three weeks after initial scan Extensive T2-FLAIR hyperintensity within both thalamocapsular regions (A), pons (B), and midbrain (C), seeming to affect the right side more so than the left. Strong post-contrast T1 enhancement affecting both cerebral peduncles (D), with the right side extending more inferiorly to the anterior midbrain (E). Arrows pointing to foci of post-contrast enhancement (D and E) and associated oedema (A, B, and C).

PET-CT

PET-CT shown in Figure 4 demonstrated avid uptake centred in both thalami (SUV_max_: right = 25, left = 20.4), which on the right appeared to extend towards the pons, with heterogeneous uptake in the cerebellum corresponding to previous MRI findings. There were no enlarged fluorodeoxyglucose (FDG)-avid cervical, thoracic, abdominal, or pelvic lymph nodes. The mediastinal blood pool reference SUV_max_ was 2.5, and the liver reference SUV_max_ was 3.4. Overall, the appearances supported an active neoplastic or highly inflammatory process at these sites. No extracranial FDG-avid disease was identified, leaving no accessible target for biopsy. 

**Figure 4:**
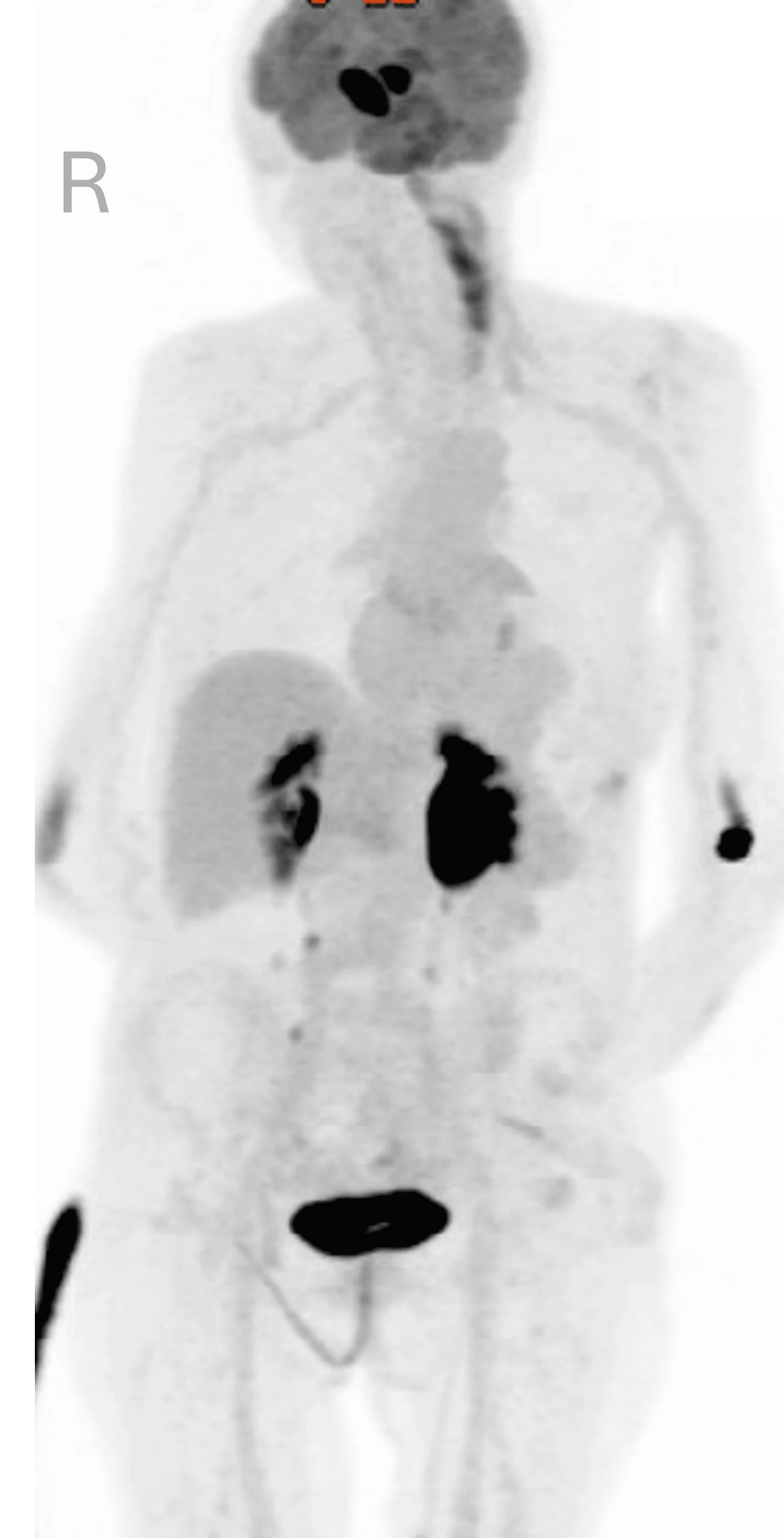
18F-fluorodeoxyglucose (FDG)-positron emission tomography-computed tomography (PET-CT) imaging An attenuation-corrected FDG study was performed from skull vertex to upper thigh. The image was taken in coronal view. Avid uptake centred in both thalami (SUV_max_ right = 25, left = 20.4; the mediastinal blood pool reference SUV_max_ is 2.5), corresponding to disease sites. No extra-cranial sites of FDG-avid disease identified for biopsy. Letter R has been added to orient to the patient's right. SUV_max_: Maximum standardised uptake value.

Neuro-oncology MDT decision and outcome: difficulty in establishing a tissue diagnosis

The case was discussed at the neuro-oncology multidisciplinary team (MDT) meeting, and a referral was made to neurosurgery. The lesion was centred on the midbrain and pons, and neurosurgical assessment concluded that the risk of stereotactic biopsy at this site was prohibitive. A brainstem trajectory carries a recognised risk of haemorrhage and new permanent neurological deficit, including cranial nerve palsy and hemiparesis, with a small but appreciable risk of death. Due to the extent of the patient's disease and her performance status, the procedure was judged unlikely to be safely achievable or to alter management even if diagnostic. PET-CT had identified no accessible extracranial target. In the absence of a tissue diagnosis, the MDT based its assessment on the convergent radiological findings, including progressive FLAIR signal change, increasingly conspicuous diffusion restriction, and a contrast-enhancement pattern typical of lymphoma, together with the patient's prior lymphoma history and the negative infective, autoimmune, and systemic disease workup. On this basis, isolated CNS relapse of DLBCL was presumed to be the most likely diagnosis, despite the absence of histopathological confirmation. The patient's performance status was assessed as ECOG 4, and she was therefore considered unsuitable for chemotherapy. The patient's history is outlined chronologically in Table 4.

**Table 4:** Timeline of key clinical events and interventions Timeline of the patient's course from initial diagnosis of diffuse large B-cell lymphoma in 2014, through completion of first-line therapy and sustained complete remission, to this admission in May 2025 and subsequent palliative outcome. Hospital days are numbered from the day of presentation. Serial MRI findings, the investigations undertaken for alternative causes, and multidisciplinary team input are shown in sequence to illustrate the evolution of imaging findings and the diagnostic interval. CNS: central nervous system; CT-NCAP: computed tomography of the neck, chest, abdomen and pelvis; DLBCL: diffuse large B-cell lymphoma; MRI: magnetic resonance imaging; PET-CT: positron emission tomography-computed tomography; T2-FLAIR: T2-weighted fluid-attenuated inversion recovery.

Time Point	Event
2014	Diagnosed with DLBCL.
February 2015	Completion of 6 cycles of chemotherapy treatment.
April 2015	In complete metabolic remission.
February 2016	12 months post-treatment CT scan; no disease progression.
October 2019	Discharged from haematology clinic; remained in complete remission
Hospital day 1	Presentation with 15 days' history of chorea, mania-like symptoms.
Hospital day 2	First MRI: non-contrast MRI head; non-specific T2-FLAIR hyperintensity centred on the midbrain and pons with some extension into the cerebral peduncles. No restricted diffusion seen.
Hospital days 2-14	Treated empirically for presumed infective encephalitis with intravenous acyclovir, ceftriaxone, and amoxicillin. Panel of investigations including serological testing, lumbar puncture and CT-NCAP was performed.
Hospital day 20	Second MRI: non-contrast MRI head performed due to lack of clinical improvement. Scan demonstrated more extensive T2-FLAIR hyperintensity extending also into the basal ganglia bilaterally.
Hospital day 23	Third MRI: contrast-enhanced MRI head performed. Areas of new restricted diffusion principally in deep thalamic regions concerning for possible CNS lymphoma relapse.
Hospital day 24	PET-CT scan showing no extracranial sites of disease available for biopsy.
Hospital days 25-30	Neuro-oncology, neurosurgery and haematology input; high-risk location for biopsy, not suitable for active treatment. Recommendation for palliative discharge and weaning oral dexamethasone course for symptomatic management.
Hospital day 44	Discharged on palliative pathway.
59 days post-discharge	Patient died at home.

Management and outcome

Dexamethasone 8 mg twice daily was commenced, with an initial improvement in symptoms. However, given her performance status and frailty, the MDT determined that she was not a suitable candidate for CNS-penetrant chemotherapy. Following detailed discussions with the family encompassing prognosis, the rationale for not pursuing curative treatment, and the goals of care, she was discharged to a nursing home on a palliative pathway with a weaning course of dexamethasone and best supportive care.

## Discussion

This case illustrates how, in the absence of a tissue diagnosis, serial MR imaging became the principal driver of diagnostic reasoning in secondary CNS lymphoma. The initial non-contrast study showed T2-FLAIR hyperintensity predominantly involving the midbrain and pons, with more limited change in the thalamocapsular regions and no restricted diffusion, an appearance not typical of lymphoma, and one interpreted at the time as a possible inflammatory or infective rhombencephalitis. It was only with progression of oedema and the appearance of new, patchy restricted diffusion on repeat non-contrast imaging 2.5 weeks later, followed by the demonstration of discrete foci of uniform, avid enhancement within an area of extensive FLAIR change on contrast-enhanced imaging, that the overall picture shifted towards CNS lymphoma relapse. This trajectory illustrates a key potential radiological pitfall: a single non-contrast study showing no restricted diffusion should not, on its own, be used to exclude CNS lymphoma in a patient with a prior haematological malignancy, and should instead prompt short-interval repeat imaging where the clinical picture remains unclear.

Intracranial CNS lymphoma can mimic other conditions due to its highly variable imaging presentation. Some differential diagnoses include high-grade glioma, metastases, and infectious and granulomatous diseases [10]. Due to this, diagnosis of secondary CNS relapse is often delayed, conferring a poor prognosis. In the short time from index imaging to the contrast-enhanced MRI and subsequent MDT review, she had declined from independent ambulation at presentation to an ECOG performance status of 4. The MDT reflected that, given her age, frailty, and the aggressive disease trajectory, she may not have been a candidate for treatment even at initial presentation.

CNS relapse of DLBCL carries a poor prognosis, with median overall survival consistently reported at approximately three to four months across large prospective and population-based cohorts [11,12]. This leaves little margin for diagnostic delay. A shorter interval from first neuroimaging to confirmed diagnosis has been associated with improved overall and progression-free survival [13], against a reported median diagnostic interval of 47 days [14]; both figures derive from cohorts of primary CNS lymphoma rather than CNS relapse of systemic DLBCL, and their applicability to the present setting is uncertain. In this case, the interval from presentation to the contrast-enhanced MRI, which established CNS lymphoma as the leading diagnosis, was 22 days. Although below the reported median, this interval nonetheless encompassed marked clinical deterioration and radiological progression, illustrating how rapidly CNS lymphoma may evolve. Where biopsy is not feasible, short-interval repeat MRI with contrast may allow more characteristic imaging features to emerge and support a working diagnosis sooner than continued observation, permitting earlier consideration of empirical treatment for lymphoma based on the working diagnosis.

A factor contributing to diagnostic delay in this case was the prolonged interval between initial systemic treatment and CNS relapse. The majority of CNS relapses in DLBCL occur within the first two years following completion of first-line therapy [11], and a long disease-free interval can reasonably, but incorrectly, reduce clinical suspicion for lymphoma as a working diagnosis. Late CNS relapse occurring years after initial systemic remission is well documented [15], and this case serves as a reminder that prior haematological malignancy should remain an active consideration in the differential regardless of remission duration. It is also notable that this patient had received intrathecal methotrexate as part of her original treatment, a strategy intended to reduce CNS relapse risk but one that is now recognised to be of limited efficacy [16].

CNS relapse risk in DLBCL is commonly estimated using the CNS-IPI (Central Nervous System International Prognostic Index), a six-point score derived by Schmitz et al. [2] from the standard IPI variables of age over 60, elevated lactate dehydrogenase, advanced (III-IV) Ann Arbor stage, ECOG performance status above 1, and more than one extranodal site, with renal and/or adrenal involvement added as a sixth factor. Patients are stratified into low-risk (0-1), intermediate-risk (2-3), and high-risk (4-6) groups, with two-year rates of CNS disease of 0.6%, 3.4%, and 10.2%, respectively. In the present case, the patient's CNS-IPI score was 2, placing her in the intermediate-risk group. This figure requires careful interpretation in the context of late relapse. The CNS-IPI was derived and validated against a two-year endpoint in cohorts in which the median time to CNS relapse was under eight months and approximately 90% of CNS events occurred within the first two years of diagnosis [2]. It is a baseline stratification tool designed to identify patients who warrant CNS-directed prophylaxis at the time of initial treatment rather than an estimate of lifetime risk of relapse. No validated model currently predicts the risk of CNS relapse of DLBCL beyond this early window.

The principal limitation of this case is the absence of histological confirmation, which remains the diagnostic gold standard in CNS lymphoma. Biopsy was considered too high risk given the lesion location and extent of disease and was, in any case, judged unlikely to alter management given the patient's performance status. The diagnosis therefore rests on the radiological evolution, the clinical context of prior DLBCL, and the absence of findings supporting an alternative cause on serological testing. None of these is individually specific for CNS lymphoma, and alternative diagnoses cannot be excluded with certainty.

Non-invasive sampling has limited sensitivity. CSF cytology carries a well-recognised false-negative rate in parenchymal CNS lymphoma, where leptomeningeal shedding of tumour cells is limited, and flow cytometry improves diagnostic yield but is insufficiently sensitive to exclude the diagnosis when negative [17]. In the present case, the cerebrospinal fluid sample was acellular and therefore non-diagnostic, providing evidence neither for nor against the diagnosis. These constraints reflect a broader diagnostic challenge: where biopsy is not feasible, a proportion of patients must be managed on radiological and clinical grounds. Cases of this kind illustrate the value of early neuro-oncology multidisciplinary involvement in establishing a working diagnosis and documenting the basis on which it was reached.

Work is ongoing to develop CSF biomarkers that may complement imaging for the diagnosis of CNS involvement by lymphoma, including the MYD88 L265P mutation [18], CXCL13 [19], and the interleukin 10: interleukin 6 ratio [20]. It should be noted, however, that these markers have been investigated and validated predominantly in the context of primary CNS lymphoma rather than CNS relapse of systemic DLBCL specifically, and their diagnostic utility in this specific setting remains less well established. None were tested in this case; further prospective validation in the CNS relapse of DLBCL population will be needed before they can be relied upon to support or substitute for radiological diagnosis in patients for whom biopsy is not feasible.

## Conclusions

This case describes a presumed isolated CNS relapse of DLBCL occurring years after initial systemic remission, with an atypical clinical constellation and early imaging that did not conform to the classic radiological pattern of lymphoma. Although histological confirmation was not obtained, the radiological evolution and clinical context were consistent with this diagnosis. As a single unconfirmed case, it cannot establish how frequently CNS relapse presents in this manner, but it illustrates a pattern with practical implications for investigation. When CNS relapse is a possibility, a single non-contrast MRI is not a sufficient basis on which to exclude it. Diffusion restriction, although a classical feature of CNS lymphoma, may be absent earlier in the disease process, and its absence does not rule out the diagnosis. Where the clinical picture is ambiguous and CNS relapse is included in the differential diagnosis, short-interval repeat MRI with contrast should be considered, as CNS lymphoma can progress rapidly in a matter of weeks.
